# Supplementary material for: Patterns of Intron Gain and Loss in Fungi
Source: PLoS Biol. 2004 Nov 30;2(12):e422. doi: 10.1371/journal.pbio.0020422 (PMC532390; doi:10.1371/journal.pbio.0020422)
Supplement: Table S1 — Also available at http://genes.mit.edu/NielsenEtAl/. (4.3 MB ZIP). [file pbio.0020422.st001.zip › NielsenEtAl/html/923.html]

AN7422.1.NCU05777.1.MG08270.1.FG02640.1


```
 CLUSTAL W (1.82) Multiple Sequence Alignments - Introns Inserted


Sequence 1: MG08270.1	787 aa
Sequence 2: FG02640.1	774 aa
Sequence 3: NCU05777.1	788 aa
Sequence 4: AN7422.1	697 aa
Alignment Length: 797 aa
Number Identitical Residues: 326 aa
Alignment Score (without introns) 18069


MG08270.1 	MACTHLESLS1LQPPTPAQSVYREDCTQCFDSI0DGPDGLDVCLQCF~NGGCVGERYHAK
NCU05777.1	MSCPHIGSAN1LAKPSPTDNVYREDCTQCFDSI0DDPAGLDVCLKCF~NGGCAGDRNHAK
FG02640.1 	MACPHLDLIG1LTPPDPSQSVYREDCTQCFDSI0DDPDGLDVCLQCF~NGGCAGERNHDR
AN7422.1  	----------~----------------------~---------MIYL0N---YGHR----
          	                                           :  : *    *.*    

MG08270.1 	LHHLIWSHPLVLNVRRT~RKHIDRDEPPMKMSKLAIAAETEADRYDYHTKVKCLDCESE-
NCU05777.1	LHATSRNHPLVLNIRRT~RKIIERNEPPLKMSKLAIAAETESDRYDTTLTVKCLDCGIDN
FG02640.1 	LHYALTQHPLALNIRRT~RKVVERDEPPAKMSKLAIAAETEEDRYDTALAVKCLDCNTE-
AN7422.1  	-------LPLKPFIEKT0-----REEPPHKISKLAINAETDEDRYDTSTRVVCYSCGRDD
          	        **   :.:*      *:*** *:***** ***: ****    * * .*  :.

MG08270.1 	LDKAHPKLAKVVDGVMTANTFSRKEEVKAWEQELTSCEHILTLQQDEPRQIASNELGTCS
NCU05777.1	LNKEDATIAPVIEAVMKANTFSRKEEVKAWEQELTTCEHILTMQQGEPRKIESQDLGHCS
FG02640.1 	LDRTNPKLAPMVDGILKANTFSRKEEVKAWEQELTSCEHILMLQQAPSKKIEQNDLSHCY
AN7422.1  	VGEPSGKLQSVIEGVMHATTFSKREEIKAWEQEFIPCEHTTGLIQQESKRIKSQ------
          	:..   .:  :::.:: *.***::**:******: .***   : *  .::* .:      

MG08270.1 	KCDLNENLWLCLQCGALG~CGRAQFGGVSGHSHALAHATESGHGVAVKLGSITPEGTADV
NCU05777.1	KCDLNENLWLCLECGNLG~CGRAQFGGVSGNSHGLAHATETKHGVAVKLGSITPEGTADV
FG02640.1 	ACDLAENLWLCIECGNLG~CGRKQMGGVDGNSHALAHSNESGHGVAVKLGSITPEGTADI
AN7422.1  	-----------------G1CGRSQFGGTGGNSHGLAHFNATSHAVAVKLGSITADGSADI
          	                 * *** *:**..*:**.*** . : *.*********.:*:**:

MG08270.1 	YCYSCDEERVDENLGHHLANYGINIAEREKTEKSLTEMQIEQNLRWEFSMTTEDGKELKP
NCU05777.1	YCYNCDEERIDDNLGAHLANWGIILAEREKTEKSLTEMQIEQNLKWEFSMTTEDGKELTP
FG02640.1 	YCYKCDDERVDDKLGEHLNHWGIVLAERQKTEKSLTEMQIEQNLKWDFSMTTEDGKELKP
AN7422.1  	YCYKCNEERIDPDLATHLYHWGIDLAGREKTEKSLMEMQVEQNLKWDFLMTTEDGQDLTP
          	***.*::**:* .*. ** ::** :* *:****** ***:****:*:* ******::*.*

MG08270.1 	LFGPGLTGLKNLGNSCYLASIIQCLFDMDAFQKRYG-QVTQELPGIQDPAADLETQLRKI
NCU05777.1	VFGPGLTGLKNLGNSCYLASILQCLYDLPSFQQRYYSDKLGPLPSVSDPAEDLETQLRKL
FG02640.1 	LFGPGLTGLRNLGNSCYLASIVQCLFDMPSFQSRYF-QPGADLPIVPEPAADLETQLRKV
AN7422.1  	VFGAGLTGLSNLGNSCYLSSVVQCLFDLPEFQRRYY-HPNEDPPLAETPAADFETQLRKL
          	:**.***** ********:*::***:*:  ** **  .     *    ** *:******:

MG08270.1 	GDGLLSGRYSKPDSDVVASEHSAELPYQKGLQPSMLKHLVGRGHEEFSTMRQQDAFELLQ
NCU05777.1	GDGLLSGRYSKSDADVFASEHTPNVPYQKGLQPSMFKHLMGRGHEEFSTMRQQDAFELLQ
FG02640.1 	ADGLLSGRYSKPDTEIAR----EAATHQKGLAPAMLKHLIGRGHEEFSTMRQQDALEFLQ
AN7422.1  	ADGTLSGRYSRPDDKTVASPKPQEIRHQKGLAPSMFKHLIGRDHPEFSTMRQQDAFEFLL
          	.** ******:.* .   :  .    :**** *:*:***:**.* **********:*:* 

MG08270.1 	HLIKLISRAKHPSPADDPTQDFRFILEQRLQCLGCKKVRYTTNEQDSIFIDVPLEKLPVE
NCU05777.1	HLIKLITRSKHPVELGDPTQPMRFVMEQRLQCLNCKKVRYSSNEQDSVFIDVPMEKLPPE
FG02640.1 	HLFKLITRSSH-SDGKDPTEPFRFVLEQRLQCLGCHKVKYSSNEQDNIFIDVPLEKLPRE
AN7422.1  	HVFKHVTLSKHPVGMDNPIDSFKFSIEQRLQCLKCRGVRYRLDEQDNISIPVPARRVPDS
          	*::* :: :.*.    :* : ::* :******* *: *:*  :***.: * ** .::* .

MG08270.1 	EGGETEGNTGAAYKPVTLSECLDRFTAEEIVELTCSSCGSKAGYTKRSCFKTLPQVLVVN
NCU05777.1	EEGQEPK-----YKPVTLKQCLDSLTASEVVELTCAGCGSKDGFTKRQAFKTFPDVLVVN
FG02640.1 	EGSDGPD----AYKPVSLTQCLDNFTAAEKVELTCSACGSKDGFSKQSLFKTFPDTLVVN
AN7422.1  	EGSNSDG----SFESVTLYDCLDAFTADETVDLRCAHCNSQEKFSKRSAFRTMPAELVIN
          	* .:       :::.*:* :*** :** * *:* *: *.*:  ::*:. *:*:*  **:*

MG08270.1 	ARKMTIVNWVPIKVDVPVLVNDDPYLLDNYLSQGQQPGEELLP-EDASSSSAPAFTPNPE
NCU05777.1	ARKMAVVNWVPVKIDVPVIVDDEPLNLDEYLSKGQQPDEEALPEDESAKSSAPAFVPNAE
FG02640.1 	ARKMTVVNWVPIKVDVPVLVPDKPFNLDAYLSKGLQPGEEQLP-DEPE-AKAPAFEPDAT
AN7422.1  	ARRFELINWVPTKLDIPVEVEDAPIDLSCYLSTGPGEGEDLLPEDEET----GGFEVNMD
          	**:: ::**** *:*:** * * *  *. *** *   .*: **.::       .*  :  

MG08270.1 	AVAQLEAMGFPRNRCEKALHATGNSDANTAMEWLFGHMEDPDIDEPLKLEGSGDGAGGF-
NCU05777.1	ALAMLEAMGFPRVRGEKALHATGNSDANAAMEWLFAHMDDPDIDVPLDLSASSGGAASGG
FG02640.1 	AMSQLEAMGFPYNRCARALHATGNSDANTAMEWLFGHMEDPDIDAPLDLGAQTGAAD---
AN7422.1  	TLNQLLAMGFPRVRCEKALYSTGNSDAEAAMNWLLSHMDDPDIDEPVHKKRVSEFGAN--
          	::  * *****  *  :**::******::**:**:.**:***** *:.       . .  

MG08270.1 	--TADPSSIEMLGAMGFDAKKSTKALKETSGNVERAVEWLFSHPDDDGSMED-DSAAAS-
NCU05777.1	AGAADPEKLAMLESMGLSGPRAVKALNETGGDVERAIEWLFSHPDDDGADAVEEEAPAGN
FG02640.1 	--TADPEKIETLGAMGFGAPQAKKALKETGGDLERAVEWLFSHPDDQGDDDAPAEGASS-
AN7422.1  	--EQDLAKIAQLGDMGIDDSRARKALTATGGDINRAIDWVFSHPGDE-DESSVHEPPSGN
          	    *  .:  *  **:.  :: ***. *.*:::**::*:****.*:       . .:..

MG08270.1 	-GEKPPAGKSDVPAQYQLKSIVCHKGTSIHAG2HYVAFIRKQLQQD-KPAWVLFNDEKVV
NCU05777.1	EGPKKEAGSSELPAKFQLQSIVCHKGTSIHAG2HYVAFIRKKLSED-ETSWVLFNDEKVV
FG02640.1 	-ATKEPAGSADLPATFQLRSIVCHKGTSIHAG2HYVAFIRKALGDANVPTWVLFNDEKVV
AN7422.1  	ADTSVVPGFATSPATYQLRSIICHKGASVHAG2HYVAFVRKHLPGKSGLSWVMFNDEKVV
          	   .  .* :  ** :**:**:****:*:*** *****:** *   .  :**:*******

MG08270.1 	EVVDVEEMKKFAYIYFFNRVGSS
NCU05777.1	KVVDVEEMKKTAYVYFFKRV---
FG02640.1 	EAHDVDEMRKFAYVYFFKRV---
AN7422.1  	EVDDVQEMKKFAYLYFFSRV---
          	:. **:**:* **:***.**
```
